# Notes and News

**Published:** 1894-07-21

**Authors:** 


### Notes and News.

Collected and Communicated.

The Hospital Sunday Fund Collection for 1894 has now reached the sum of £41,800.

A very good way of encouraging and stimulating local support is adopted by the Royal Hospital at Richmond. The amounts collected by boxes are published half-yearly and sent round to contributors, who thus become reminded of the needs of the hospital, and acquainted with what is being done for it. If a list of ordinary subscriptions were also added, we think this would make the system yet more effective.

The foundation-stone of the Convalescent Home for Charing Cross Hospital, which is to be erected at Oxted, near Limpsfield, was laid last week. The hospital is indebted for this great addition to its resources to the munificence of Mr. Passmore Edwards, who has offered to bear the whole expense of building, furnishing, and fitting, at an estimated cost of about £10,000. The home is to contain fifty inmates.

The Swansea General Hospital is considering a scheme which provides that if a body of workmen contribute thirty-five guineas annually they may nominate one of their number as a governor. The recommendation of the House Committee is to be considered at a special meeting, the Chairman desiring it to be understood that the committee accepted the principle of working-men representation, but that it was not quite decided upon the terms of qualification. It is announced that an anonymous gift of £1,300 had been received for the purpose of endowing one bed in the institution.

Amongst the exhibits at the Antwerp Exhibition which has excited interest and attention is the medicine chest used by Mr. Stanley in his African expeditions. Surgeon Parke's small case of instruments is also shown. Messrs. Borroughs and Wellcome show novelties for travellers at the exhibition which are likely to become popular. These are tea tabloids, made of compressed tea, each serving for one cup; one hundred tabloids cost but sixpence, a small sum to expend in testing the tabloids, which, if as good in quality as they are convenient in form, will be regarded as indispensable by travellers.

We learn from the Secretary-General of the Congress of Hygiene and Demography, to be held at Budapest in September, that the preliminary arrangements are nearly concluded, and that a brilliant success is anticipated. Representation promises to be very complete. Twenty-six governments of the public corporations, 41 universities, and 132 learned societies are all sending delegates. There will be a good exhibition of models of public sanitation works as a special feature. Special railway arrangements are being made for the convenience of members, and the official invitation tickets are soon to be issued.

The British Institute of Public Health holds a Congress from the 26th to the 31st inst. Amongst the vice-presidents are the Archbishop of Canterbury, Lord Salisbury, and Canon Vaughan. Local authorities have accepted invitations for delegates to the number of 1,500 so far. The Congress will be divided into five sections: Preventive medicine, chemistry and climatology, engineering and building, construction, sewage purification, naval and military hygiene, and other matters of public interest will all be considered and discussed. The opening meeting will be in the Guildhall. The Lord Mayor will attend in state. Prof. Smith, of King's College, will act as president.

Recent revelations of cruelties and misadventures have shown that the children in large pauper schools have but mechanical and inadequate supervision, while all who are conversant with the barrack system know that there are serious evils affecting the health, developement, and character of children who are brought up in great numbers in one building. With the view of securing a thorough investigation into pauper schools, and in the hope that it may be found possible to substitute boarding out—or when that is not possible a system of cottage homes—for the children, such as will relieve them from many of the existing disadvantages, a deputation will wait on Mr. Shaw-Lefevre to ask for a Royal Commission and to lay before him the grounds which would justify the request for an exhaustive and searching inquiry. Mr. Shaw-Lefevre has agreed to receive the deputation at half-past two on Monday, July 23rd, at the Local Government Board offices.

Contrary to the usual course of an epidemic recurring several years consecutively, the cholera now prevalent in St. Petersburg is reported to be of a more fatal nature than during its previous visitations. Further, it is said to be assuming alarming proportions, and has penetrated even into Finland. In St. Petersburg the hospitals are full, and the use of an old prison for patients is being contemplated. Carbolic acid is being distributed throughout the city, the sanitation of which is declared to be in a very bad condition. The Sanitary Commission has assembled to sit continuously whilst the epidemic prevails. In England, although no very great apprehension has yet arisen, the sanitary authorities are taking due precautions. The Local Government Board has issued a circular to these sanitary authorities, warning them that outbreaks of diarrhœa should be regarded with suspicion, and suggesting that such outbreaks should be duly notified whilst cholera was prevalent in any part of Europe, or at seaports.

